# Classic Kaposi Sarcoma: Current Treatment Strategies and Emerging Therapeutic Approaches

**DOI:** 10.3390/cancers18061008

**Published:** 2026-03-20

**Authors:** Daniela Revenko, Natali Shirron, Reut Shainer, Emily Avitan-Hersh, Alona Zer

**Affiliations:** 1 Faculty of Medicine and Surgery, University of Parma, 43126 Parma, Italy; daniela.revenko@studenti.unipr.it; 2 Fishman Oncology Center, Rambam Health Care Campus, Haifa 3109601, Israel; n_shirron@rambam.health.gov.il (N.S.); r_shainer@rambam.health.gov.il (R.S.); 3 Department of Dermatology and Skin Cancer Laboratory, Rambam Health Care Campus, Haifa 3109601, Israel; e_avitan@rambam.health.gov.il; 4 The Rappaport Faculty of Medicine, Technion—Israel Technology Institution, Haifa 3200003, Israel

**Keywords:** classic kaposi sarcoma, novel therapies, treatment

## Abstract

Classic Kaposi sarcoma (CKS) is a rare vascular neoplasm driven by infection with Kaposi sarcoma-associated herpesvirus (KSHV) and characterized by a heterogeneous geographic distribution. While the etiology of other Kaposi’s sarcoma (KS) variants is well established, the underlying mechanisms of CKS appear multifactorial, with several risk factors, among which advanced age and male sex are most significant. Due to the rarity of CKS and its often indolent clinical course, clinical trials are sparse, and current knowledge of therapeutic approaches remains limited. Most available clinical trials and practice guidelines focus on human immunodeficiency virus (HIV) -related Kaposi sarcoma. Therefore, specific recommendations for the classic form are restricted and rely on a low level of evidence, as categorized by the National Comprehensive Cancer Network (NCCN) Clinical Practice Guidelines, making disease management more challenging. Current approaches to treating CKS include both local and systemic therapies, selected based on disease stage, patient comorbidities, and individual preferences. Systemic treatment options may include immunotherapy, chemotherapy, or antiangiogenic agents. This review summarizes current management strategies and highlights emerging therapeutic approaches for CKS. It aims to support clinicians in optimizing therapeutic decision-making, including the use of novel and investigational therapies. Although several novel therapies are currently under investigation in clinical trials, significant gaps remain. Therefore, further research is needed to improve disease management.

## Simple Summary

Classic Kaposi sarcoma (CKS) is a rare vascular neoplasm associated with human herpesvirus-8 (HHV-8). It characteristically follows an indolent clinical course, typically manifesting in elderly populations of Mediterranean and Eastern European descent. While CKS often presents as localized cutaneous lesions, its management becomes increasingly complex when patients develop symptomatic systemic complications. Because of its rarity and the specific population it affects, robust prospective clinical trial data remain limited; consequently, current knowledge regarding standardized therapeutic strategies is often limited to retrospective analyses and expert consensus. Furthermore, the challenge of treating elderly patients with multiple comorbidities necessitates a personalized approach to minimize toxicity. However, the evolving understanding of tumor pathogenesis has paved the way for innovative interventions. This review provides a comprehensive, up-to-date overview of current and emerging therapeutic strategies for CKS. We discuss established treatment modalities and shift focus toward emerging approaches, including the promising roles of immunotherapy and antiangiogenic agents.

## 1. Introduction

Classic Kaposi sarcoma (CKS) is a rare vascular neoplasm with a heterogeneous clinical presentation and low incidence. It predominantly affects elderly men and most commonly presents with cutaneous lesions, while visceral or systemic involvement is relatively uncommon [1,2,3]. Despite its generally indolent course, advanced or refractory disease poses significant therapeutic challenges. Evidence is often derived from small studies, and standard treatment options are limited, particularly for patients who do not respond to standard treatments [4]. This review summarizes current treatment strategies and discusses emerging therapeutic approaches that may expand future management options.

## 2. Current Knowledge

### 2.1. Epidemiology and Risk Factors

Kaposi sarcoma (KS) is a rare multifocal angioproliferative neoplasm caused by infection with human herpesvirus 8 (HHV-8), also known as Kaposi sarcoma-associated herpesvirus (KSHV). KSHV is a double-stranded DNA virus with oncogenic potential. Four clinical forms have been described: Classic KS (CKS), which is the focus of this review; Endemic KS (African); Iatrogenic KS (organ transplant-related); and Epidemic KS (HIV-associated) [1].

CKS is predominantly seen in older men from Mediterranean, Eastern European, and Middle-Eastern populations [2]. Among developed countries, one of the highest incidences of CKS has been reported in Jewish populations in Israel [5].

CKS predominantly affects men, with male-to-female ratios of 2:1 in Italy, 5:1 in Israel [3,6] and 7:1 in the United States [2]. The incidence of CKS increases with age [3], with a mean age at diagnosis of 71 years [7]. The etiology of HIV-associated and transplant-associated KS involves both KSHV infection and impaired host immunity. In contrast, the predisposition to CKS in elderly men of Mediterranean or Jewish descent remains unclear and may be related to specific Human Leukocyte Antigen (HLA) types [8]. Other potential contributing factors include environmental, infectious, and genetic factors [9].

An increased risk of CKS has been associated with topical corticosteroid use, a history of asthma or allergies [9,10] and comorbidities such as diabetes mellitus and herpes simplex virus infection [11]. A higher incidence of gout has also been reported in patients with CKS, suggesting a possible association between the two [12].

Although most CKS cases are sporadic, rare familial clustering has been documented, suggesting a potential role for heritable factors. Genetic associations have been reported with several HLA alleles and variants in cytokine-related genes, such as *STAT4* [13,14]. Immune dysregulation associated with stromal interaction molecule 1 (STIM) deficiency has been linked to a more aggressive clinical course, and OX40 (CD134) deficiency has also been identified in patients with CKS [15,16]. Further evidence supporting a genetic contribution comes from the identification of a dominantly inherited mutation in the *BPTF* gene in two HIV-negative, unrelated families with CKS. Functional studies suggest that this mutation contributes to KSHV pathogenesis by altering oncogenic and angiogenic pathways [17].

### 2.2. KSHV Biology and Pathogenesis

KSHV, first identified in 1994, is known as the etiologic agent not only of KS but also of primary effusion lymphoma (PEL) and multicentric Castleman disease (MCD) [18]. Although the exact routes of transmission remain unclear, detection of HHV-8 in saliva and semen suggests that viral spread may occur through exchange of bodily fluids [19].

**Figure 1:**
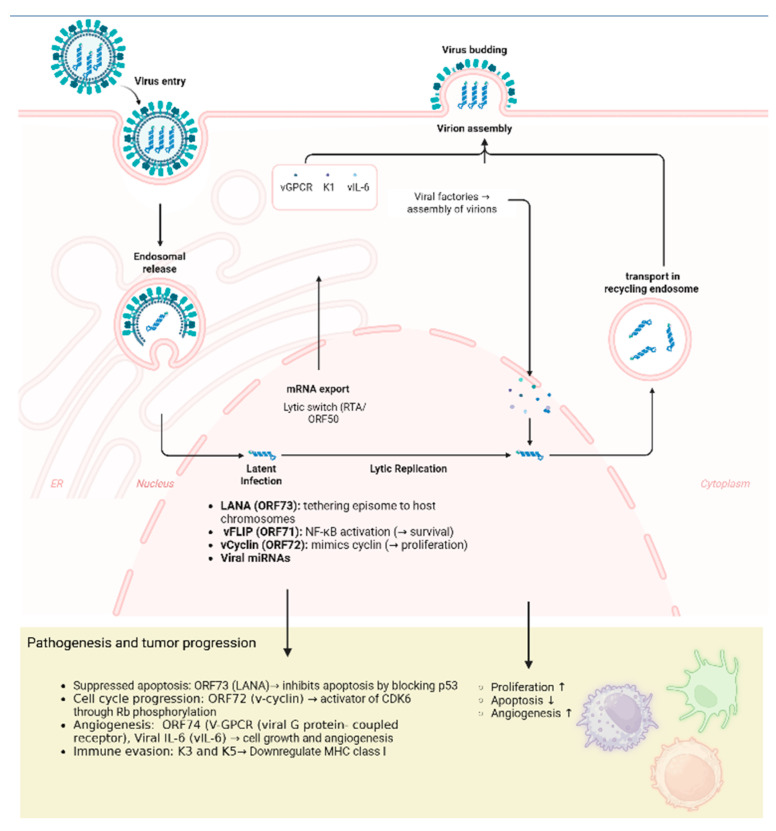
KSHV pathogenesis: from viral persistence to oncogenic development. Schematic representation illustrating the interplay between viral latent and lytic phases and host cellular machinery. The figure highlights the role of key viral proteins, including LANA, v-cyclin, and vFLIP, in cell-cycle dysregulation and inhibition of apoptosis through p53 and Rb pathways. These molecular alterations promote cellular transformation and oncogenesis, thereby contributing to the development of KS.

The molecular mechanisms underlying KSHV infection and oncogenesis are summarized in Figure 1. Following infection of target cells, primary B cells and endothelial cells, the viral capsid is transported to the nucleus, where the viral genome persists as an episome. Viral persistence and tumor development are largely mediated by the expression of latent viral genes. A key protein in this process is the latency-associated nuclear antigen (LANA; *ORF73*), which anchors the episome to host chromosomes during cell division and inhibits apoptosis through interactions with the p53 pathway. Viral FLICE-inhibitory protein (vFLIP; ORF71) activates the NF-kB signaling pathway, while viral cyclin (v-cyclin; ORF72) constitutively activates cyclin-dependent kinase 6 (CDK6), thereby driving cell-cycle progression through phosphorylation of the retinoblastoma (Rb) protein.

Beyond promoting cell survival, the virus also induces changes in the tumor microenvironment. Viral G-protein-coupled receptor (vGPCR; *ORF74*) and viral interleukin-6 (vIL-6) promote angiogenesis and inflammation. Kaposin proteins encoded by *ORF K12* promote pro-inflammatory cytokine production, enhance cellular motility and contribute to tumor progression. To evade the host immune response, KSHV encodes immune-modulatory proteins, including K3 and K5, which downregulate major histocompatibility complex (MHC) class I molecules and thereby impair immune recognition. Although latency represents the predominant state of viral persistence, KSHV can also undergo lytic reactivation, regulated by the replication and transcription activator (RTA; *ORF50*), and triggered by environmental stressors, inflammatory cytokines, or oxidative stress. These viral proteins, expressed during both latent and lytic phases, not only ensure viral persistence, survival, and immune evasion but also play a central role in tumor development and progression [20,21].

### 2.3. Clinical Manifestations

**Figure 2:**
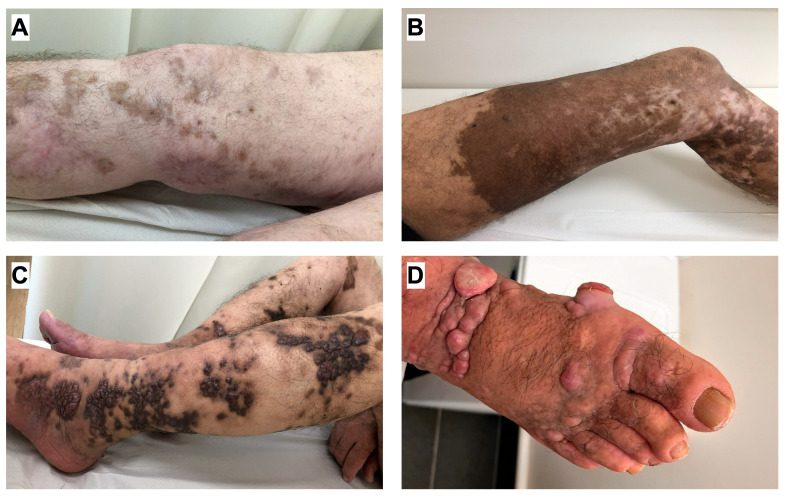
Selected clinical manifestations of CKS. (**A**) Flat, brownish patches observed in the patch stage. (**B**) Brown to violaceous plaques with multiple firm, pink-purple nodules, characteristic of the nodular stage. (**C**) Confluent, extensive patches and plaques. (**D**) Multiple large, ulcerated nodules accompanied by edema.

The clinical manifestations of CKS typically follow a chronic, indolent course. Lesions usually appear as asymptomatic, symmetric, multifocal macules, papules, plaques, or nodules (Figure 2). They are predominantly located on the lower extremities and can range in color from pale pink to purple or reddish-brown [21]. Three main clinical stages have been described: the patch stage, the plaque stage, and the nodular stage. Different morphological stages often coexist in the same patient [22].

Mucosal involvement may occasionally occur; however, visceral metastases are relatively uncommon and typically develop late in the disease course [1]. Complications such as pain, edema, ulceration, secondary infection, and lymphorrhea are relatively uncommon [23,24]. Lymphedema is a common complication and may result from lymph node involvement and/or lymphatic vessel invasion [7]. Clinically, KS skin lesions may be misdiagnosed as venous or arterial ulcers, other vascular lesions, or chronic infected wounds [24]. Compared with CKS, the epidemic, endemic, and iatrogenic forms of KS are generally more aggressive, often demonstrating faster progression and earlier visceral dissemination [24].

### 2.4. Diagnosis and Staging

The diagnosis of CKS is established through clinical evaluation and histopathological confirmation, typically obtained by skin biopsy of a representative lesion [25]. In the early stages, histopathology is characterized by proliferation of thin endothelial cells forming irregular, dilated vascular channels, often accompanied by extravasation of red blood cells. In the plaque stage, abnormal vascular structures extend into the deeper dermis, and spindle-cell fascicles become more prominent. In the nodular stage, dense spindle-cell proliferation with vascular and lymphatic-like spaces replaces the normal dermal architecture [8].

Several histologic variants of KS have been described, although their clinical significance remains uncertain. These include Pre-KS (or in situ KS), which resembles early patch-stage lesions or anaplastic KS, an aggressive form with a high mitotic index, marked atypia, and occasional necrosis [22].

Immunohistochemistry plays a crucial role in the diagnostic evaluation of KS. LANA-1 immunostaining is the most reliable method for detecting HHV8, producing a characteristic punctate nuclear staining pattern that helps distinguish KS from histologically similar lesions [22]. The presence of HHV8 is generally consistent across epidemiologic variants and is not associated with patient age, sex, or tumour recurrences [26].

Accurate assessment of KS lesions is essential for determining disease stage, which guides therapeutic decision-making and prognosis [25]. Unlike HIV-associated KS, no universally accepted staging system exists for classic, iatrogenic, or endemic KS. Therefore, additional prognostic factors, such as tumor burden and risk of visceral involvement, should be considered. Because visceral disease is rare in CKS, the extent of cutaneous involvement remains the most reliable indicator of disease severity [25].

Historically, CKS staging was based on Krigel’s classification, but more recent reviews suggest that Brambilla’s classification provides a more accurate framework [25]. In this system, stage I (macronodular) includes small macules confined to the lower extremities; stage II (infiltrative) involves larger plaques still limited to the lower extremities; stage III (florid) includes multiple plaques and nodules in the lower extremities; and stage IV (disseminated) includes lesions extending beyond the lower extremities [26]. As no standardized staging system exists for non-HIV KS, European consensus guidelines classify patients into three categories: localized non-aggressive, locally aggressive, and disseminated disease. For CKS, endemic KS, and post-transplant KS, staging should be tailored to the clinical presentation and rate of lesion progression [27]. In most cases of CKS, advanced imaging modalities are rarely required due to the frequently limited cutaneous involvement. However, NCCN guidelines recommend clinical photography as an essential component of the initial workup and follow-up. While chest X-ray is indicated to screen for thoracic involvement, CT is reserved for specific clinical scenarios, such as onset of new gastrointestinal or pulmonary symptoms or to investigate abnormalities found during routine monitoring. Findings on chest CT may include ill-defined nodules and pleural effusions. Furthermore, MRI has shown a more accurate evaluation of KS lesions, which demonstrate increased signal intensity on T-2 weighted imaging. Finally, whole body FDG PET\CT is a valuable tool for identifying extracutaneous extensions, but it remains less sensitive in detecting lesions within the mucosal surfaces of the digestive tract [28,29].

## 3. Therapeutic Strategies

Optimal management of CKS requires an integrated approach, with careful consideration of the role of each therapeutic modality in different clinical scenarios [30]. Because KS is driven by latent KSHV infection and typically presents as a multifocal disease, treatment of a single lesion does not prevent the development of additional lesions. Therefore, aggressive surgical resection, as used for other soft-tissue sarcomas, is generally not indicated. Watchful observation may be appropriate for immunocompetent patients without symptoms. Symptomatic lesions can be effectively managed with topical therapies, radiotherapy, or surgical excision. In cases of florid or disseminated disease, or in infiltrative stages with rapid progression or resistance to local treatments, systemic therapy is usually the preferred treatment option [27,30].

### 3.1. Local Therapy

#### 3.1.1. Radiotherapy

KS is a radiosensitive tumor [31], and radiotherapy remains a cornerstone in the management of localized CKS, achieving overall response rates of 88–100% [27,31,32,33], with a complete response rate of 58–93% [27,32], and symptomatic relief in 95–100% of patients [27,32]. Radiotherapy (RT) has also been shown to be effective for both mucosal and cutaneous lesions [32]. Several RT regimens can be used for palliation and local disease control, and randomized data suggest that hypo-fractionated regimens are as effective as conventional fractionation [34].

Brachytherapy represents an alternative RT modality, demonstrating high response rates in small lesions, especially in elderly patients or in cases where cosmetic outcomes are a concern [31]. Radiotherapy is generally well tolerated, with adverse effects such as dry desquamation, hyperpigmentation, and lymphedema, which often improve during follow-up [32,35].

#### 3.1.2. Topical Agents

In contrast to the high response rate achieved with RT, topical agents offer a non-invasive treatment option for patients with limited disease. However, their clinical efficacy varies and is supported by heterogeneous levels of evidence. Topical imiquimod 5% cream acts as a Toll-like receptor 7 agonist, enhancing local immune activation, inhibiting angiogenesis, and inducing endothelial apoptosis [36,37]. It has demonstrated antitumor activity in cutaneous KS, with complete responses reported in approximately one-third of patients and partial responses in another third. Several studies have also reported no recurrence after one year of follow-up [38]. In addition, an open-label phase I–II study in HIV-negative patients reported a response rate of 47% with good tolerability [39]. Reported adverse effects were generally mild and included local erythema, pruritus, and occasional flu-like symptoms [38,40].

Topical alitretinoin 0.1%, typically applied twice daily, has been studied mainly in HIV-positive patients; evidence supporting its efficacy in CKS remains limited. Nevertheless, a case report described near-complete remission of lesions after 3.5 months of treatment [40]. Alitretinoin acts by binding to retinoid receptors and regulating gene expression pathways involved in cellular proliferation, differentiation, and apoptosis [41,42].

Several additional topical agents have been investigated for cutaneous KS, although evidence remains limited and heterogeneous.

#### 3.1.3. Surgical Treatment

While local intervention may be necessary in selected cases, surgical treatment plays a limited role in the management of KS and is used primarily for diagnostic purposes or for the removal of lesions located at anatomically critical sites [43]. A retrospective study of non-HIV-infected adults found no significant difference in outcomes between patients treated with wide excision and those managed with other therapeutic modalities [44].

#### 3.1.4. Intralesional Chemotherapy and Immunomodulatory Agents

Intralesional chemotherapy involves direct injection of cytotoxic agents into KS lesions, allowing high local concentrations while minimizing systemic toxicity. Intralesional vincristine has been reported as a safe and effective treatment for nodular lesions, with complete response rates of up to 76% [45]. Similarly, bleomycin combined with electroporation (electrochemotherapy) has demonstrated complete response rates of 65–89% [46]. In addition to cytotoxic agents, perilesional interferon (IFN) has been shown to promote regression of KS lesions. This effect may be enhanced when combined with interleukin-2, as the two agents act synergistically to amplify local immune responses [47]. However, the use of intralesional chemotherapy or immunotherapy is limited to patients with small localized lesions for symptomatic control, and its use in routine clinical practice remains relatively uncommon.

#### 3.1.5. Cryotherapy and Laser

Cryotherapy with liquid nitrogen is a simple and effective treatment option for cutaneous KS, especially for lesions that are unresponsive to systemic therapy or associated with poor cosmetic outcomes. In one series, 63% (19/30) of patients achieved complete remission without recurrence [48]. Combination therapy with cryotherapy and topical imiquimod has also demonstrated efficacy in limited cutaneous KS [49].

The long-pulse Nd:YAG laser, which penetrates deeper dermal layers than many other laser systems, represents a safe and effective option for papulonodular or deeper lesions, particularly those located over osseous prominences [50].

Based on current evidence, radiotherapy remains the gold standard for symptomatic localized lesions, given its strong clinical validation and high response rates. Other local therapies, such as intralesional chemotherapy, may be appropriate for smaller lesions, whereas cryotherapy and laser therapy are supported by more limited clinical data in CKS and should therefore be considered in selected cases.

### 3.2. Systemic Therapy

KS is considered a systemic disease driven by chronic KSHV infection. Although curative treatment is not achievable, therapy aims to achieve durable disease control by reducing the number and size of cutaneous and visceral lesions. Available systemic therapies are summarized in Table 1.

#### 3.2.1. Cytotoxic Chemotherapy

##### Liposomal Anthracyclines

Pegylated liposomal doxorubicin (PLD) is the most established first-line systemic therapy for KS [13]. Doxorubicin exerts its cytotoxic effects by intercalating into DNA and inhibiting topoisomerase II, thereby causing DNA strand breaks and apoptosis. Liposomal encapsulation of conventional doxorubicin with a polyethylene glycol (PEG) coating enables drug accumulation within KS lesions by exploiting their abnormal, highly permeable vasculature [51]. This formulation also reduces exposure to healthy tissues, thereby minimizing systemic toxicity compared with conventional anthracyclines.

Clinically, PLD achieves high response rates with a favorable safety profile and is typically administered intravenously at 20–30 mg/m^2^ every two to three weeks [52]. In a randomized trial, PLD demonstrated superior efficacy and tolerability compared with the bleomycin-vincristine combination in KS, with an overall response rate of 46% with PLD versus 25% with the combination [53].

PLD is primarily associated with palmar-plantar erythrodysesthesia and mucositis as dose-limiting toxicities [54]. Other adverse effects include mild myelosuppression and reduced cardiotoxicity compared with conventional doxorubicin; however, current National Comprehensive Cancer Network (NCCN) guidelines recommend an echocardiogram prior to initiating treatment, followed by periodic reassessment. Less common adverse effects include alopecia, nausea, and vomiting [55,56].

##### Taxanes

Taxanes, particularly paclitaxel, are microtubule-stabilizing agents that disrupt the dynamic reorganization of the microtubule network required for mitosis. Importantly, their clinical activity has been specifically validated in non–HIV-associated KS populations across independent studies [57,58]. Unlike other systemic treatments, for which evidence is often extrapolated from HIV-positive cohorts, the efficacy of taxanes in CKS is supported by dedicated prospective phase II clinical studies. In the largest CKS-specific cohort to date [57], weekly paclitaxel (80–100 mg) achieved complete responses in 15.9%, partial responses in 63.7%, and stable disease in 13.6% of patients, resulting in disease control in more than 90% of cases. Nab-paclitaxel, an albumin-bound formulation of paclitaxel, avoids the need for steroid premedication and is generally better tolerated in elderly patients with CKS [59]. Docetaxel has been less extensively studied but has demonstrated objective response rates of up to 40% [57]. Neutropenia represents the main dose-limiting toxicity, along with fatigue and peripheral neuropathy [58].

Overall, taxanes combine antimitotic and antiangiogenic mechanisms [60] and demonstrate consistent clinical activity, making them valuable systemic options for non-HIV-associated KS.

A critical limitation in CKS management is the absence of head-to-head randomized controlled trials comparing systemic therapies. Consequently, clinical consensus is largely based on retrospective analyses and data derived from HIV-associated cohorts.

Within this framework, PLD remains the preferred first-line systemic therapy for CKS, due to its favorable safety profile and well-established efficacy. While taxanes are typically reserved for cases of PLD resistance or intolerance. Other chemotherapeutic agents, including vinblastine, gemcitabine, vinorelbine, etoposide, and bleomycin, may be considered second-line alternatives but are generally not recommended as first-line treatments [52].

#### 3.2.2. Antiangiogenic Agents

Angiogenesis plays a crucial role in KS pathogenesis, driven by KSHV-induced secretion of VEGF, PDGF, and FGF, which promote vascular permeability, edema, and tumor progression, processes that are essential for tumor growth [61]. Lenalidomide and pomalidomide, thalidomide analogues, bind cereblon (a ubiquitin ligase complex protein), leading to reduced proliferation and induction of apoptosis [62]. In addition, they inhibit angiogenesis by downregulating VEGF and other pro-angiogenic cytokines [63]. In HIV-negative KS, pomalidomide achieved a response rate of 80% in a phase I/II trial and is now FDA-approved for KS in both HIV-positive (after ART failure) and HIV-negative patients, with generally good tolerability, but potential hepatotoxicity and myelotoxicity [64]. Lenalidomide has shown more modest activity, with response rates ranging from none to partial response rates up to 40% of patients [65].

mTOR inhibitors, such as sirolimus and temsirolimus, reduce VEGF secretion [66] and are effective in post-transplant KS, where switching from calcineurin inhibitors to sirolimus is common [67]. However, sirolimus has not demonstrated meaningful benefit in classic KS, as its immunosuppressive effects may outweigh its antitumor activity [68].

Sorafenib and pazopanib are oral multi-kinase inhibitors with antiangiogenic and antiproliferative activity, designed to target VEGF-driven signaling pathways. Pazopanib, which also targets fibroblast growth factor receptors (FGFRs), is approved for renal cell carcinoma and soft tissue sarcomas; however, clinical evidence in KS remains limited, with only a single reported complete response [69].

Bevacizumab is a recombinant humanized monoclonal antibody that binds soluble VEGF, thereby blocking VEGF-receptor interaction and inhibiting endothelial proliferation and angiogenesis [70]. In a phase II study of KS, bevacizumab achieved an overall response rate of 31% [71].

The combination of pegylated liposomal doxorubicin (PLD) with bevacizumab achieved a 56% response rate and median progression-free survival of 6.9 months, although no clear advantage over PLD monotherapy [72].

Despite modest activity and the potential development of resistance, antiangiogenic agents remain a promising therapeutic strategy, particularly in combination regimens. The bidirectional crosstalk between angiogenic pathways and the immune system provides a strong rationale for combining antiangiogenic therapies with immunotherapy, an approach already approved in several other malignancies and potentially applicable to KS [73].

#### 3.2.3. Immunotherapy

Evidence from early KS lesions, which display dense immune infiltrates and abundant pro-inflammatory cytokines, suggests that the immune response plays an active role in tumor development [74]. In a phase II trial of 18 elderly patients with progressive classic KS, nivolumab plus ipilimumab achieved a response rate of 78–93%. Toxicity was manageable, with grade 3–4 adverse events occurring in 22% of patients [75]. In a multicenter, single-arm, phase II study that included patients with classic and endemic KS, pembrolizumab achieved an overall response rate of 71%, with durable responses and an acceptable safety profile [76].

In classic KS, responses have also been reported with low-dose interferon, administered at 3–5 million IU subcutaneously three times weekly [77]. However, this treatment is now rarely used and supporting clinical data remain limited.

Currently, no direct comparative trials have been conducted between immunotherapy and antiangiogenic agents in CKS. Indirect evidence suggests that immunotherapy may provide higher response rates and more durable remissions, while maintaining an acceptable safety profile that requires monitoring for immune-related adverse events. Antiangiogenic agents remain an important therapeutic option, particularly for patients who are ineligible for immunotherapy or have contraindications to immune checkpoint blockade. Overall, immunotherapy appears to be associated with higher response rates and more durable disease control than antiangiogenic agents in CKS, although both approaches have distinct toxicity profiles and clinical indications.

**Table 1:** Recommended Systemic Therapies for Classic Kaposi sarcoma *.

Line of Therapy	Drug Name	ORR	Common Side Effects	References
First-line therapy	Pegylated liposomal doxorubicin	60–80%	Neutropenia, nausea, asthenia	[52,78]
Second-line therapy for refractory cKS	Paclitaxel **	75–80%	Neutropenia, alopecia, peripheral neuropathy, myelosuppression, myalgias	[58]
Antiangiogenic agents	Pomalidomide	70–80%	Neutropenia, hepatotoxicity, thrombosis	[64]
Immunotherapy	Nivolumab + ipilimumab	87%	Hepatotoxicity, GI toxicity, pneumonitis	[75]
	Pembrolizumab	62–71%	Pruritus, fatigue, arthralgia	[76,79]

* Recommendations are adopted in accordance with the NCCN guidelines. ** If paclitaxel is intolerant, nab-paclitaxel is recommended.

## 4. Clinical Trials and Novel Therapies

A significant unmet need remains for novel, well-tolerated oral therapies for CKS, and treatment should be individualized according to the patient’s performance status and overall clinical condition. Consequently, the search for innovative therapeutic strategies is ongoing, and several emerging approaches are currently being evaluated in clinical trials. Key ongoing clinical trials in CKS are summarized in Table 2.

An ongoing trial of the oral CDK4/6 inhibitor abemaciclib demonstrated a response rate of 84% in a small cohort of both HIV-positive and HIV-negative patients with KS, possibly through inhibition of v-cyclin mediated CDK6 activation [80].

In a phase I trial, efineptakin alfa (NT-17), a long-acting interleukin-7 (IL-7) fusion protein designed to enhance host T-cell-mediated immunity against KSHV-driven tumors, achieved an overall response rate of 42.9%. Notably, all responders were HIV-positive, although the study also included HIV-negative patients [81].

The clinical trial NCT04305691 is investigating Ixazomib, a proteasome inhibitor widely used in the treatment of multiple myeloma, which is currently being evaluated in a phase II trial for KS.

NHS-IL-12 (clinical trial ID NCT04303117), a fusion protein composed of interleukin-12 and NHS76, has shown immune activation and antitumor activity in preclinical models. It is currently being investigated in KS as monotherapy or in combination with M7824, a bifunctional anti-PD-L1/TGF-β fusion protein.

Angiogenesis plays a central role in KS pathogenesis. Hypoxia-inducible factor 1α (HIF-1α), a key regulator of tumor growth, also enhances HHV-8 gene expression and lytic replication. Digoxin is currently being evaluated as a potential HIF-1α inhibitor in the phase II multicenter KADIG-01 trial. In addition, digoxin has demonstrated antiviral activity against other herpesviruses, such as cytomegalovirus and herpes simplex virus [82].

A missense mutation in the *BPTF* gene has been identified as a monogenic hereditary factor predisposing to KSHV-induced oncogenesis, highlighting *BPTF* as a potential therapeutic target for future drug development [16].

Finally, the phase II trial (ID NCT02799485) is evaluating a recombinant EphB4-HSA fusion protein, which has demonstrated antiangiogenic activity in preclinical studies, suggesting another potential therapeutic strategy for KS [83].

## 5. Future Perspectives

Looking ahead, the therapeutic landscape for CKS is shifting toward a “chemo-sparing” paradigm. The primary unmet clinical need remains the development of oral administration, low-toxicity agents suitable for long-term use in an elderly population with multiple comorbidities. Although ongoing trials for agents such as abemaciclib and ixazomib are promising, future research should prioritize CKS-specific cohorts, rather than relying on data extrapolated from HIV associated KS. From mechanistically prespective, targeting the tumour microenvironment and the angiogenic-hypoxic axis (e.g., with inhibitors such as digoxin) may represent a more sophisticated approach than conventional cytotoxic treatment. In addition, identification of genetic predispositions, such as BPTF mutation, may pave the way for personalized medicine.

Ultimately, the goal is to transform CKS into a manageable chronic disease while preserving quality of life through individualized treatment approaches. Collectively, these approaches have the potential to expand the available treatment options for CKS. Continued research is essential to strengthen the evidence base and improve disease management.

Another important unresolved issue in the management of classical Kaposi sarcoma (CKS) is the optimal duration of systemic therapy. Prospective data addressing treatment duration are lacking, and most recommendations are extrapolated from studies in HIV-associated KS. In clinical practice, systemic chemotherapy, most commonly pegylated liposomal doxorubicin or paclitaxel, is typically administered until maximal response or a response plateau is achieved, or until unacceptable toxicity. Several studies have used approximately 6 cycles administered every 2–3 weeks [78], although the number of cycles is frequently individualized according to response and tolerability. Because CKS often follows a chronic and relapsing course, treatment may also be interrupted after response and re-introduced at relapse, supporting a strategy of intermittent disease control rather than continuous therapy. Further prospective studies are needed to define the optimal duration and sequencing of systemic treatment.

## Figures and Tables

**Table 2:** Selected and ongoing notable clinical trials in cKS.

Drug	Mechanism of Action	Study ID	Status	Study Design	End Point (Primary Goal)
Abemaciclib	Blocks cell cycle by binding to CDK4/6, by preventing phosphorylation of RB	NCT04941274	Recruiting	Phase I/II study assessing the safety and efficacy of abemaciclib in participants with previously untreated or treated KS.	Safety and tolerability and ORR
efineptakin alfa (NT-I7)	An IL-7 agonists, binds to IL-7 receptor on CD-4+ and CD-8 cells, stimulating their proliferation	NCT04893018	Terminated	Phase I Study of for Patients with or without Infection with HIV	Adverse events
Ixazomib	Inhibits 20S subunit of proteosome, causing accumulation of misfolded proteins, thus causing apoptosis in malignant cells	NCT04305691	Recruiting	phase II trial, determine the overall response rate and safety of ixazomib in participants with Kaposi sarcoma	ORR
NHS-IL12 or in combination with M7824	Recombinant immunocytokine, enhances immune activation by delivering IL-12 in tumour site	NCT04303117	Recruiting	This is a Phase I/II study assessing the safety and efficacy of NHS-IL12 or in combination with M7824 for patients with advanced KS	Safety, tolerability and activity
EphB4-HSA	Inhibiting EphB4-ephrin-B2 signaling pathway, crucial for tumor angiogenesis and vascular remodelling	NCT03993106	Recruiting	phase II trial studies toxicity and clinical response	Clinical response and toxicity

## Data Availability

No new data were created or analyzed in this study. Data sharing is not applicable to this article.

## Abbreviations

The following abbreviations are used in this manuscript: CDK6: Cyclin-dependent kinase 6, CKS: Classic Kaposi sarcoma, HHV-8: Human herpesvirus 8, HIV: Human immunodeficiency virus, HLA: Human leukocyte antigen, IFN: Interferon, KSHV: Kaposi’s sarcoma-associated herpesvirus, LANA: Latency-associated nuclear antigen, MCD: Multicentric Castleman disease, MHC: Major histocompatibility complex, ORR: Overall response rate, ORF: Open reading frame, OX40: Tumor necrosis factor receptor superfamily member 4 (CD134), PEL: Primary effusion lymphoma, PLD: Pegylated liposomal doxorubicin, Rb: Retinoblastoma protein, RTA: Replication and transcription activator, STIM1: Stromal interaction molecule 1, v-cyclin: Viral cyclin, vFLIP: Viral FLICE-inhibitory protein, vGPCR: Viral G-protein-coupled receptor, vIL-6: Viral interleukin-6
